# THE IMPACT OF SMALL INTESTINAL BACTERIAL OVERGROWTH ON THE GROWTH OF CHILDREN AND ADOLESCENTS

**DOI:** 10.1590/1984-0462/2020/38/2018164

**Published:** 2020-01-13

**Authors:** Alexandre Neves da Rocha Santos, Ana Cristina Fontenele Soares, Ricardo Palmero Oliveira, Mauro Batista de Morais

**Affiliations:** a Universidade Federal de São Paulo, São Paulo, SP, Brazil.

**Keywords:** Intestine, small, Child, Adolescent, Breath tests, Lactulose, Growth, Intestino delgado, Criança, Adolescente, Testes respiratórios, Lactulose, Crescimento

## Abstract

**Objective:**

To evaluate the association between small intestinal bacterial overgrowth (SIBO) and weight and height impairment in children and adolescents with gastroenterology diseases.

**Methods:**

Observational and retrospective study. All 162 patients aged less than 19 years old who underwent breath test in search of SIBO between 2011 and 2016 were studied. Breath test was collected after the intake of 10 grams of lactulose. The concentration of hydrogen and methane was measured for 180 minutes after the beginning of the test by 12i QuinTronMicroLyzer device.

**Results:**

SIBO was identified in 51 (31.5%) patients. There was no difference between the age of those with (mean=8.7y.o; 25^th^ and 75^th^ percentile: 4.6 and 11.3) and without (mean=7.9y.o 25^th^ and 75^th^ percentile: 4.8 and 12.2) SIBO (p=0.910). There was no association between gender and SIBO (male 26.3% vs. female 36.3%, p=1.00). A lower median of height-for-age Z score (mean=-1.32; 25^th^ and 75^th^ percentile: -2.12 and -0.08 vs. mean=-0.59; 25^th^ and 75^th^ percentile: -1.57 and 0.22; p=0.04) was demonstrated in children with SIBO when compared with children without it. There was no difference between the BMI-for-age Z score of patients with (mean=-0.48) and without SIBO (mean=-0.06) (p=0.106). The BMI of patients with SIBO (median=15.39) was lower than of those without it (median=16.06); however, the statistical analysis was not significant (p=0.052). The weight-for-age Z score was lower in patients with SIBO (mean=-0.96) than in those without SIBO (mean=-0.22) (p=0.02)

**Conclusions:**

Children and adolescents with SBIO associated with diseases of the gastrointestinal tract have lower weight and height values.

## RESUMO

**Objetivo:**

Avaliar a existência de associação entre sobrecrescimento bacteriano no intestino delgado (SBID) e comprometimento de peso e estatura em crianças e adolescentes com doenças do aparelho digestivo.

**Métodos:**

Estudo observacional e retrospectivo em ambulatório de gastroenterologia pediátrica. Foram incluídos todos os 162 pacientes com idade inferior a 19 anos que realizaram teste respiratório para pesquisa de SBID entre 2011 e 2016. O teste respiratório foi realizado após ingestão de dez gramas de lactulose. Foram determinadas as concentrações de hidrogênio e metano em aparelho 12i QuinTron MicroLyzer até 180 minutos após o início do teste respiratório.

**Resultados:**

SBID foi caracterizado em 51 (31,5%) dos 162 pacientes. Não houve diferença na idade das crianças com (mediana=8,7 anos; percentil 25–75: 4,6–11,3) e sem (mediana=7,9 anos; percentil 25–75: 4,8–12,2) SBID (p=0,910). Não se observou associação entre SBID e sexo (masculino 27,4% e feminino 36,6%; p=0,283). O escore Z da estatura-idade nos pacientes com SBID (mediana=-1,32; percentil 25–75: -2,12—0,08) foi menor (p=0,040) do que naqueles sem SBID (mediana=-0,59; percentil 25–75: -1,57–0,22). Na comparação do escore Z de índice de massa corpórea-idade não foi observada diferença entre os grupos com (média=-0,489±1,528) e sem (média=-0,067±1,532) SBID (p=0,106). Nos pacientes com menos de 10 anos de idade, o escore Z de peso-idade foi menor nos pacientes com SBID (média=-0,968±1,359) do que nos sem SBID (média=-0,223±1,584) (p=0,026).

**Conclusões:**

Crianças e adolescentes com SBID associado a doenças do trato gastrintestinal apresentam menores valores de peso e estatura.

## INTRODUCTION

Small intestinal bacterial overgrowth (SIBO) is characterized by an abnormal increase in the amount of bacteria in the lumen of the small intestine and/or the presence of atypical microbiota in this portion of the gastrointestinal tract. SIBO may manifest with symptoms such as flatulence, steatorrhea, chronic diarrhea, abdominal distension, chronic abdominal pain, among others, or may be asymptomatic.^1-3^

SIBO is traditionally thought to occur when there are anatomical and intestinal motility abnormalities.^2-5^ It may also be found in functional gastrointestinal disorders such as irritable bowel syndrome, functional abdominal pain, and functional constipation.^6-13^ SIBO is still associated with poverty and may favor nutrient malabsorption as part of environmental enteropathy.^14-17^

Height impairment has been noted in children with SIBO living in underdeveloped countries when interpreting the concentrations of hydrogen (H_2_) and methane (CH_4_) in the lactulose breath test.^16^ Previous studies using only H_2_ to interpret the test results did not show a height deficit in children with asymptomatic SIBO.^17^ Growth impairment may be a justification for SIBO treatment regardless of symptoms.

The gold standard for SIBO diagnosis is the analysis of microbiota in jejunal content.^1-3^ However, it is an invasive method, which is also expensive and difficult to perform. An alternative for the diagnosis of SIBO is the respiratory test after ingestion of lactulose or glucose.^1-4^ This test analyzes the concentrations of H_2_ and CH_4_ in breath samples. H_2_ and CH_4_ production is common due to anaerobic fermentation in the large intestine. In the presence of SIBO, this production also occurs in more proximal portions of the gastrointestinal tract.^1-4^

Considering the above, the aim of this study was to evaluate if there is an association between SIBO and weight and height impairment in children and adolescents with digestive tract diseases. In the present study, we evaluated the weight and height values of patients treated for digestive tract diseases and who underwent H_2_ breath testing after lactulose administration for SIBO research.

## METHOD

The design of this study was observational and retrospective. The study was conducted at the pediatric gastroenterology outpatient clinic of the Escola Paulista de Medicina, Universidade Federal de São Paulo (UNIFESP). Data were collected from patients, regardless of the reason for the consultation, under the age of 19 years who underwent lactulose breath testing between August 2011 and January 2016.

The following information was collected from the electronic medical record: gender, date of birth, date of first visit, weight and height at first visit, diagnostic hypothesis, test date, weight and height with a maximum interval of two months before or after the breath test.

Anthro Plus 3.2.2 software provided by the World Health Organization (WHO) (available at http://www.who.int/childgrowth/software/en/), was used to measure body mass index (BMI)- age and height-age of all patients, as well as the weight-age Z-score of patients under ten years of age only.

The H_2_ and CH_4_ breath test in exhaled air was performed according to the protocol adopted in the clinical laboratory of the pediatric gastroenterology discipline of Escola Paulista de Medicina, UNIFESP. The exams were performed in the morning after fasting for six to eight hours. Initially, oral hygiene with 0.5% chlorhexidine was performed. Then, the fasting expired air sample was collected. After this step, a solution containing 10 grams of lactulose diluted in 100 mL of water was administered orally. Samples of exhaled air were collected at 15, 30, 45, 60, 90, 120, 150 and 180 minute intervals after lactulose ingestion.^4,13-17^

The no rebreathing valve set up device designed by QuinTron Instrument Company Inc. (Menomonee Falls, Wisconsin, USA) was used to collect exhaled air samples. The device has a valve that allows air to pass in one direction only. Exhaled air samples were kept in a polyethylene syringe attached to a three-way tap. Ambient air samples were collected at the same location as the breath test. The measurement of H_2_ and CH_4_ concentrations in exhaled air samples was performed by gas chromatography using the QuinTron MicroLyzer 12i model (QuinTron Instrument Company - Milwaukee, Wisconsin, USA). Results were expressed in parts per million (ppm). SIBO was characterized when there was an increase in H_2_ (≥20 ppm) and/or CH_4_ (≥10 ppm) concentration in relation to the fasting value until the sample collected at 60 minutes after lactulose ingestion. Non-H_2_ producers were considered as those patients who did not present a minimum elevation of 10 ppm H_2_ in exhaled air after lactulose ingestion in any of the samples.^12-17^

For the analysis of the results, the SigmaPlot 12.0 software (Systat Software Inc., USA) and Epi-Info version 6 (Centers for Disease Control and Prevention, USA) were used, using setting 5% as the level to reject the null hypothesis. The chi-square test was used for the analysis of categorical variables. Numerical variables were evaluated by the Mann-Whitney test or Student’s t-test, depending on the distribution of each variable. The findings of a previous study were considered in order to estimate the sample size.^16^ Thus, considering a 5% alpha error, 80% power, a difference between the mean height-age Z score averages of 0.4 and a standard deviation of 0.9, it was estimated that 47 patients with SIBO would be needed (Systat Software Inc., USA).

This study was approved by the Research Ethics Committee (CEP) of UNIFESP under CAAE number 62059116.7.0000.5505.

## RESULTS

We evaluated 162 patients, 91 (56.2%) were male. The age ranged from six months to 19 years. 51 (31.5%) presented SIBO. The distribution of patients and the diagnostic hypotheses that led to the SIBO research are presented in Table 1. The statistical study did not showed higher positivity, with a statistically significant difference in SIBO in any of the diseases that motivated the request for the H_2_ breath test.

**Table 1:** Age, gender and diagnostic hypothesis of children and adolescents who underwent lactulose breath hydrogen and methane breath testing according to the presence of bacterial overgrowth in the small intestine.

	With SIBO (n=51)	Without SBID (n=111)	p-value
Age (years)	8.7 (4.6–11.3)	7.9 (4.8–12.2)	0.910^a^
Sex			
Male	25 (27.4%)	66 (72.6%)	0.283^b^
Female	26 (36.6%)	45 (63.4%)	
Diagnostic hypothesis			
Cow Milk Protein Allergy	1 (12.5%)	7 (87.5%)	0.189^b^
Suspected underweight and / or height	8 (61.5%)	5 (38.5%)	
Intestinal constipation	15 (33.3%)	30 (66.6%)	
Chronic Diarrhea	8 (25.8%)	23 (74.2%)	
Abdominal pain	6 (23.1%)	20 (76.9%)	
Gastroesophageal Reflux Disease	2 (33.3%)	4 (66.6%)	
Lactose intolerance	2 (18.2%)	9 (81.8%)	
Other	9 (40.9%)	13 (49.1%)	

SIBO: Bacterial overgrowth in the small intestine; ^a^Mann-Whitney test (median and 25–75 percentiles in parentheses); ^b^Chi-square test.

Among the 162 patients, 24 (14.8%) were classified as non-H_2_ producers. Among these 24 non-H_2_-producing patients, four were diagnosed with SIBO based on increased exhaled air CH_4_ concentration after lactulose ingestion.

Table 2 shows the height-age and BMI-age Z-scores of the 162 patients included in the study. The height-age Z score of children with SIBO was lower than that of children without SIBO, and the difference was statistically significant. When comparing the BMI-age Z score, no difference was observed between the groups with and without SIBO.

**Table 2:** Z-score of height-age and body mass-age index according to the presence of bacterial overgrowth in the small intestine.

	With SIBO (n=51)	Without SIBO (n=111)	p-value*
Z-score of height-age	-1.32 (-2.12—0.08)	-0.59 (-1.57–0.22)	0.040^a^
BMI-age Z score	-0.48±1.52	-0.06±1.53	0.106^b^

SBID: Bacterial overgrowth in the small intestine; BMI: body mass index; ^a^Mann-Whitney test (median and 25-75 percentiles in parentheses); ^b^Student’s t-test (mean ± standard deviation)

Table 3 compares the weight-age Z score of 103 children under ten years old. Weight-age Z-scores were lower in SIBO patients.

**Table 3:** Weight-age Z-score of patients under ten years of age according to the presence of bacterial overgrowth in the small intestine.

	With SIBO (n=30)	Without SIBO (n=73)	p-value*
Weight-age Z score	-0.96±1.35	-0.22±1.58	0.026

SBID: Bacterial overgrowth in the small intestine; *Student’s t-test (mean ± standard deviation).

Table 4 presents the area under the concentration curve of H_2_ and CH_4_ obtained in the breath test, used as lactulose fermentation indicators. The production of H_2_ and CH_4_ was higher in patients with SIBO in the period between zero and 60 minutes and between 60 and 180 minutes.

**Table 4:** Area under the curve of hydrogen and methane concentration, in parts per million / minutes, of the lactulose breath test of patients with or without small intestinal bacterial overgrowth during the first 60 minutes, between 60 and 180 minutes and in the period total of the test.

		With SIBO (n=51)	Without SIBO (n=111)	p-value*
H_2_	0–60 min	735.0 (382.5–1237.5)	292.5 (120.0–570.0)	<0.001
	60–180 min	4830.0 (2880.0–7290.0)	2145.0 (1260.0–3210.0)	<0.001
	0–180 min	5775.0 (3622.5–8842.5)	2512.5 (1447.5–3810..0)	<0.001
CH_4_	0–60 min	202.5 (0.0–1230.0)	0.0 (0.0–375.0)	0.006
	60–180 min	975.0 (0.0–3750.0)	0.0 (0.0–1005.0)	0.003
	0–180 min	1402.5 (0.0–4770.0)	30.0 (0.0–1545.0)	0.006

SIBO: Bacterial overgrowth in the small intestine; *Mann-Whitney test (median and 25–75 percentiles in parentheses).

## DISCUSSION

In the present study, it was demonstrated that children with digestive tract diseases diagnosed with SIBO have lower values of weight and height than those without SIBO. It has also been shown that the production of H_2_ and CH_4_ in exhaled air in the breath tests of patients with SIBO is higher, not only in the small intestine but also in the colonic phase of the breath test. SIBO was most often found to be associated with other digestive tract diseases such as cow’s-milk protein allergy, constipation, chronic diarrhea, gastroesophageal reflux disease, and lactose intolerance (Table 1).

To our knowledge, to date, no articles have been published that correlate the presence of weight-to-height deficit in pediatric patients with SIBO associated with digestive tract disease. Height-weight deficit has been described^15,17^ in children with SIBO associated with unfavorable environmental conditions, who may have environmental enteropathy or minimal enteric dysfunction.^15,17-19^ In these patients with asymptomatic SIBO it is debatable whether or not drug treatment should be performed. ^18^ The ideal would be to change of environmental conditions to a situation with good health conditions. Antimicrobial treatment was performed in one study with children from a community in the São Paulo Metropolitan Region.^18^ In this context, the presence of weight and height deficit, as observed in the present study, could be an additional justification for the treatment of asymptomatic SIBO with antibiotic therapy. The therapeutic effectiveness, based on the breath test, of combining metronidazole and sulfame-thoxazole-trimethoprim for two weeks for the treatment of SIBO associated with unfavorable environmental conditions has been demonstrated.^18^ In the literature, other therapeutic options include other antibiotics such as rifaximin, although a clinical trial published in 2011 showed that only 20% of patients treated with chronic abdominal pain and SIBO had normalized breath testing. In the same study, there was no difference in symptom improvement between the placebo group and the rifaximin group.^20^

Considering that there is an abnormality in the small intestine microbiota in SIBO, some authors include SIBO in the broad concept of dysbiosis and thus suggest the use of probiotics. However, to date there is no evidence to justify the use of probiotics in the treatment or prevention of SIBO.^3,21^

The results of the present study linking SIBO with weight-height deficit may be considered as an additional justification for performing SIBO drug treatment. It is noteworthy that, in this study, the individual Z-score values of patients with and without SIBO were used to characterize the nutritional repercussions of SIBO on anthropometric data. Had it been chosen to consider nutritional deficit as a categorical variable (cutoff below -2 standard deviations of the Z-score), these differences would not have been identified (data not shown). Thus, more sensitive measures were used in the comparison of the groups. This had already been observed in a study conducted more than two decades ago in our service.^22^

In 2009, a European consensus suggested that, in the breath test for the diagnosis of SIBO, glucose should be used as the preferred substrate.^23^ In our experience, lactulose was always used for SIBO research using the breath test.^13-18,22^ In one of these articles^17^, both glucose and lactulose tests were performed, showing that H_2_ production with glucose was very low, which presumably underestimated the prevalence of SIBO in the study population.^17^

In this context, in another consensus published in 2017, it was recommended to use both lactulose and glucose as a substrate to be used in a study on SIBO using the breath test.^4^ The use of CH_4_ together with H_2_ in the exhaled breath test increases the sensitivity of the breath test for the diagnosis of SIBO. This was demonstrated in articles published by our research group.^15,22^

In patients with SIBO, higher production of H_2_ and CH_4_ has been evidenced in the presumably colonic fermentation phase.^16-18,22^ Our study also reached this result (Table 4). This is suggestive that intestinal microbiota abnormalities in patients with SIBO are not restricted to the small intestine. Thus, in our opinion, the elevation of H_2_ and CH_4_ in exhaled air for diagnosis of SIBO should be restricted to the first 60 minutes of the breath test, considering that the orocecal transit time is shorter in children than in adults.

This study is limited as it was retrospective, with only one test per patient. Confirmation of the relationship between SIBO and weight-to-height deficit should be performed in future projects, in which anthropometric follow-up is performed after SIBO treatment. Other limitations are the two-month interval between obtaining anthropometric data and the breath test of H_2_ and CH_4_ in exhaled air, the variability in the previous disease duration that led to the diagnosis of SIBO and the lack of information in the medical records about environmental conditions of the patients.

In conclusion, it was observed that the production of H_2_ and CH_4_ in children with SIBO is higher during the colonic period of the breath test. And, responding to the objective of the present study, it was found that pediatric patients with SIBO associated with gastrointestinal tract diseases have lower height values and those under ten years also have lower weight values when compared to patients without SIBO.
